# Impact of Kinesio Taping on Oral Feeding and Swallowing Functions: Acoustic Analysis of Swallowing Sounds in Late Preterm Infants—A Randomized Clinical Trial

**DOI:** 10.3390/children12030369

**Published:** 2025-03-15

**Authors:** Nilay Comuk Balci, Deniz Anuk Ince, Ayşe Ecevit, Balkar Erdoğan, Ilknur Ezgi Doğan, Ozden Turan, Aylin Tarcan

**Affiliations:** 1 Department of Physiotherapy and Rehabilitation, Faculty of Health Sciences, Ondokuz Mayıs University, 55270 Samsun, Türkiye; 2 Department of Pediatrics, Division of Neontology, Faculty of Medicine, Baskent University, 06790 Ankara, Türkiye; denizanuk@yahoo.com (D.A.I.); ayseecevit@yahoo.com (A.E.); drozdentr@yahoo.com (O.T.); 3 KuartisMED (MC), Technology Transfer Office (TTO), Middle East Technical University, 06800 Ankara, Türkiye; info@kuartismed.com (B.E.); aylintarcan@yahoo.com (A.T.); 4 Department of Physiotherapy and Rehabilitation, Faculty of Health Sciences, Baskent University, 06790 Ankara, Türkiye; ilknurezgidogan@gmail.com

**Keywords:** swallow, preterm, newborn, Kinesio taping, sucking

## Abstract

**Background/Objectives**: Feeding difficulties in late preterm infants are a major factor contributing to prolonged hospitalization and re-admission. Early support for the sucking and swallowing muscles may accelerate their maturation, facilitating safe and early discharge. This study aims to evaluate the effects of the Kinesio-taping technique on feeding muscles and assess feeding and swallowing function in late preterm infants through the acoustic analysis of swallowing sounds. **Methods:** Seventy-four late preterm infants (mean gestational age 35.30 ± 0.81 weeks) were randomly assigned to either a Kinesio-taping group or a control group. A single physiotherapist applied Kinesio taping to support the masseter and hyoid muscles, using a facilitatory technique to enhance muscle function. The Kinesio taping was removed two days after its application. The amount of milk intake, the time for milk intake, oxygen saturation during milk intake, the number of days required for transition to full oral feeding, the length of hospital stay, the duration of oxygen requirement, the maximum number of rhythmic swallows, and the heart rate during milk intake were recorded using a digital stethoscope before and two days after Kinesio-taping application. The collected data were assessed through acoustic analysis. **Results:** No statistically significant differences were observed between the Kinesio-taping and control groups regarding milk intake amount, feeding duration, oxygen saturation during feeding, the transition time to full oral feeding, the length of hospital stay, or the duration of oxygen support (*p* > 0.05). However, a significant difference was found between the groups in the maximum number of rhythmic swallows during feeding and the heart rate during milk intake (*p* < 0.05). **Conclusions:** The application of the Kinesio-taping technique showed no adverse effects on preterm infants in the NICU during the feeding skills intervention. The assessment of acoustic analysis revealed a significant difference in the maximum number of rhythmic swallows and heart stabilization during feeding in the Kinesio-taping group. Further studies are warranted, incorporating different application types and techniques with larger sample sizes, especially among preterm infants with an early gestational age in the NICU, to stabilize the suck and swallow muscles.

## 1. Introduction

With advancements in medical science, an estimated 13.4 million babies were born prematurely in 2020. In 2019, approximately 900,000 children died due to complications related to preterm birth [1]. However, complications such as prolonged hospital stays, mechanical ventilation, and gastrointestinal, neurological, and cardiorespiratory immaturity can contribute to developmental and feeding issues in preterm infants. Nutrition is crucial for both parents and clinicians, playing a vital role in infant development. Problems in sucking, swallowing, and breathing coordination cause apnea, bradycardia, oxygen desaturation, and fatigue during feeding in premature infants [2,3]. Oral feeding requires high levels of energy and may prolong the infants’ hospital stay. A feeding tube is typically used in infants born before the 34th gestational week. After reaching 34 weeks of postmenstrual age (PMA), oral feeding is preferred over the feeding tube. Swallowing maturation and coordination between swallowing and respiration are crucial for safe and effective oral feeding in preterm infants. Feeding difficulties in late preterm infants are a major factor contributing to prolonged hospitalization and readmissions. Supporting the sucking and swallowing muscles during the developmental period to improve sucking–swallowing coordination can accelerate the maturation process, facilitating the safe discharge of preterm infants [4,5,6]. Increasing sensory input by the Kinesio-taping (KT) method facilitates or inhibits the stimulation of the muscle, resulting in improved coordination of the target muscle mass [7]. According to Dr Kenzo Kase, when the tape placement direction is from the origin of the muscle toward the insertion, it stimulates the muscle, and if it is from the insertion toward the origin, it inhibits the muscle [7]. The contraindication of the KT method includes taping on sites of acute infection, open wounds, deep vein thrombosis, malignancy, and severe allergy [7]. There are KT-related studies on children with torticollis, developmental delay, cerebral palsy, and swallowing difficulties [8,9,10,11,12]. In a single study, KT application was used to support swallowing in a preterm infant at the 28th gestational week due to dysphagia at postmenstrual week 40, and successful results were obtained [3,13]. The ingesting and swallowing process comprises four distinct phases: the oral phase, the initiation of the swallowing reflex, the pharyngeal phase, and the esophageal phase. The suprahyoid muscle, comprising the geniohyoid, mylohyoid, digastric, and stylohyoid muscles, assumes a crucial role in facilitating the swallowing function during the pharyngeal phase [14,15]. KT is employed to enhance muscle activation, and it is associated with heightened motor unit activation, indicative of increased muscle strength [16]. Therefore, it has been suggested that KT stimulates muscle activation by adding an extra load to the suprahyoid muscles during swallowing, which can be particularly beneficial for individuals with dysphagia [17].

A systematic review of relevant studies [18] found that implementing oral motor therapy for preterm infants in the NICU leads to positive outcomes. According to the ESPGHAN Committee on Nutrition and Invited Experts, non-nutritive sucking (NNS) before initiating oral feeding has been linked to a reduced time to achieve full oral feeding and shorter hospital stays (level of evidence: 3) [19]. Wen-Si Ni et al. [20] applied sensory stimulation around the mouth and whole-body pressure to premature infants born before 34 weeks and weighing between 1000 and 2000 g, starting 24 h after birth. As a result, the therapy group transitioned to oral feeding earlier, had shorter hospital stays, and exhibited a lower incidence of extrauterine developmental delay compared to the control group. Hwang et al. [21] and Arora et al. [22] investigated the effects of the premature infant oral motor intervention (PIOMI), developed by Beckman, which aims to activate muscle contraction and enhance strength by improving the functional response to pressure, movement, and control of the lips, cheeks, jaw, and tongue. Their findings demonstrated that infants receiving PIOMI showed greater improvements in feeding performance than the control group.

In this study, our aim was to support the sucking and swallowing muscles in infants born between 34 0/7 and 36 6/7 weeks of gestation, utilizing KT application to enhance their oral feeding performance. The objective was to address feeding problems, mitigate feeding risks, and expedite feeding maturation, comparing the results of an acoustic analysis of swallowing sounds with a control.

## 2. Materials and Methods

### 2.1. Study Design and Participants

**Figure 1:**
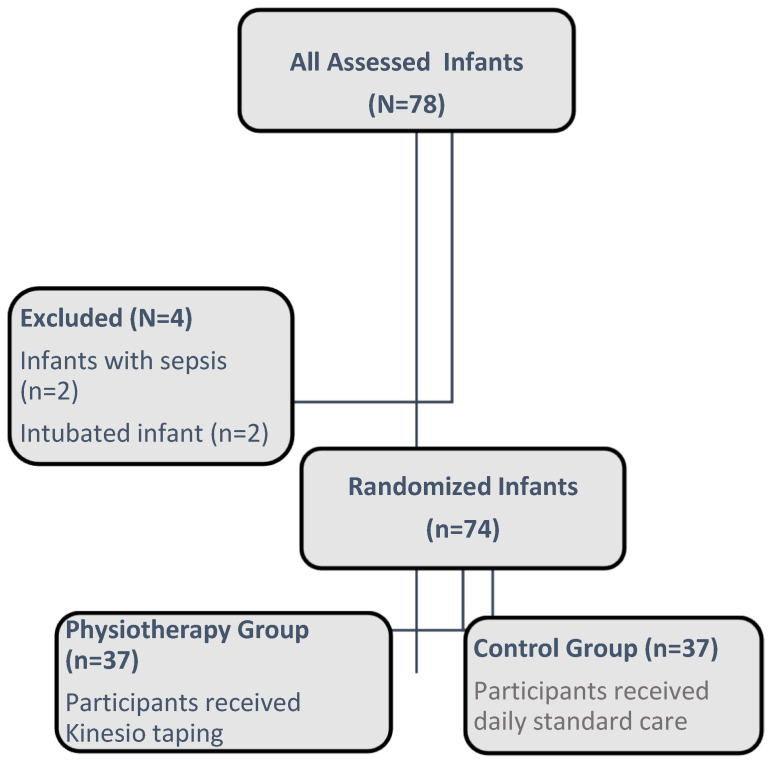
Study flow diagram.

This prospective, randomized controlled study was conducted with 74 late preterm infants, ranging in gestational age from 34 0/7 to 36 6/7 weeks. The participants were admitted to the neonatal intensive care unit (NICU) or followed up in the first few days with their mothers on the maternity ward, all hospitalized at Baskent University Faculty of Medicine, Ankara, between January 2018 and December 2018. The 74 late preterm infants were assigned to two groups according to the date of birth. We evaluated the feeding process by applying KT to 37 infants born as late preterm infants in the first group and evaluated 37 infants without KT application in the second group. Late preterm infants were assigned to either the physiotherapy group or the control group, as shown in Figure 1. The randomization sequence was generated using the computerized R program (version 3.5.1 software). Infants with intraventricular and intracranial hemorrhage, multiple congenital anomalies, hypoxic–ischemic encephalopathy, necrotizing enterocolitis, tracheoesophageal fistula, diaphragmatic hernia, respiratory distress, or hydrocephalus were excluded from the study.

### 2.2. Procedure

Infants admitted to the NICU or those followed up in the first few days with their mothers on the maternity ward were evaluated for their initial feeding both before and after the KT application on the first day. Additionally, the intake quantity in the feeding process on the second day was assessed. The amount of milk intake (mL), milk intake rate (mL/min), sucking rhythm, oxygen saturation, heart rate, and evaluation of the swallowing function were recorded. To evaluate swallowing, we recorded swallowing sounds on a digital voice recorder (Sony recorder) by placing a digital stethoscope (digital stethoscope ds32a) diaphragm in the area of the hyoid bone in the under-chin area of the infants for two minutes during the feeding session. The swallowing sounds were recorded with a digital stethoscope and evaluated with the Audacity 1.3.14 program, which is an open-source software, and the swallowing sounds were labeled using the program’s interface according to the measurements taken for two minutes, and the time indexes of each swallow were marked with the labeling. From the time indices obtained as a result of the labeling, the maximum number of rhythmic swallows were determined by calculating the time intervals between successive swallows [23]. The swallowing sounds were used for assessing feeding maturation in preterm infants, basing feeding performance on data collected during audio recordings. The swallowing sounds show the rhythmic swallows and number of swallows of the infants. Various parameters, such as the total number of swallows, maximum number of rhythmic swallows, and resting intervals were generated for each assessment [23].

**Figure 2:**
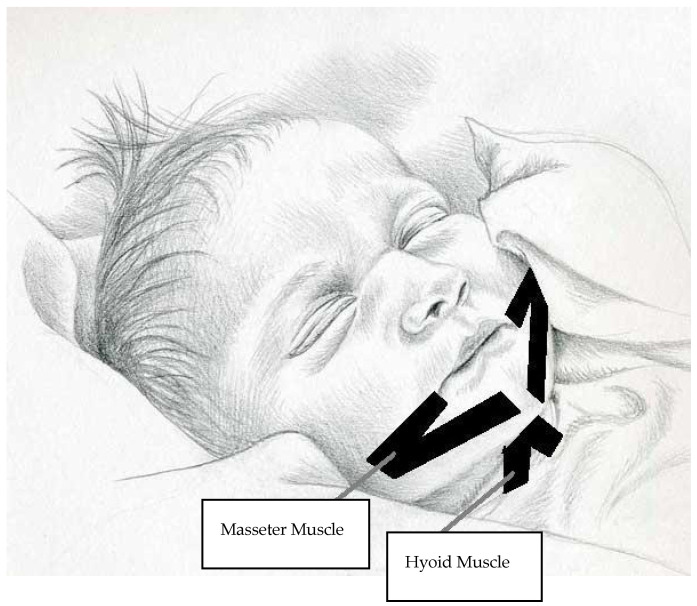
Kinesio-taping application.

In order to support sucking and swallowing, the masseter and hyoid muscles (located under the chin area) were supported with the KT technique. We applied a maximum of 10% tension in the form of Y taping on the masseter muscle for sucking and on the hyoid muscles for swallowing (Figure 2) [24]. The application of KT was carried out by a physiotherapy expert with proficiency in its usage, and the facilitatory method was used in KT application. Taping is associated with negligible adverse effects, which generally include minor skin irritation or pain while removing the tape. In order to avoid skin irritation and pain, care was taken to ensure that the band remained on the body for 72 h and to remove it with the help of glycerin.

The tape remained attached for 24 h in this area. Infants were evaluated (1) before the tape was attached 24 h after the infant’s birth, (2) at the third hour after the tape was attached, and (3) at the infant’s 48th hour. In the control group, we evaluated the feeding process in the same way: (1) at the 24th hour, (2) 3 h following feeding, and (3) at the 48th hour.

The other main outcome measures of the study were the rhythmic swallow measures and the amount of milk intake. The secondary outcome measures of the study were the time for milk intake, the heart rate during milk intake, oxygen saturation during milk intake, the number of days for transition to full oral feeding, the number of days in the hospital, and the number of days of taking oxygen.

All the procedures were carried out under the principles outlined in the Declaration of Helsinki. Additionally, all protocols were approved by the Ethics Committee of Baskent University (KA18/36, 1 December 2017). Informed parental consent was obtained from the family of the infants.

### 2.3. Sample Size Calculation

The sample size was calculated using PASS 2005 software (NCSS, Kaysville, UT, USA), and we found that 30 subjects were required for one group to achieve 90% power with a 5% type 1 error. To account for a potential 20% dropout rate, we recruited at least 37 subjects for each group, aiming to maintain 90% power in the study. A sample size calculation powered for the rhythmic swallow measures was the main outcome measure.

### 2.4. Statistical Analysis

The normal distribution analysis of the data was examined using visual and analytical methods (Shapiro–Wilk test). Because all the numeric variables showed non-normal distribution, non-parametric tests were used. Descriptive data are shown using medians and the 25th and 75th percentiles for the non-normally distributed variables. Differences between the KT and control groups were analyzed using the Chi-square test for categorical variables and the Mann–Whitney U test for numeric variables. The Friedman test was used to compare the difference within groups, and, if the test result was significant, the Wilcoxon signed-rank test was used for post hoc comparison with Bonferroni correction (statistical significance was set at *p* < 0.0167 only for the post hoc comparison). All statistical analyses were conducted with IBM SPSS Statistics 26.0 (SPSS Inc., Chicago, IL, USA), with the alpha equal to 0.05 [25].

## 3. Results

Seventy-four late preterm infants were enrolled in the study, with 37 in the KT group and 37 in the control group. No statistical differences were observed between the two groups regarding gestational age, birth weight, gender, maternal age, or twin pregnancy (*p* > 0.05) (Table 1).

No statistically significant differences were found between the KT and control groups regarding the amount of milk intake, the time for milk intake, oxygen saturation during milk intake, the number of days for transition to full oral feeding, the number of days in the hospital, or the number of days taking oxygen (*p* > 0.05). We found a significant difference between the groups in terms of the total number of swallows during milk intake and the heart rate during milk intake (*p* < 0.05) (Table 2). In terms of complications, no adverse effects related to KT were observed. No skin irritation or pain during the intervention or while removing the tape was seen in either group.

## 4. Discussion

This study investigated the effects of KT application in late preterm infants, focusing on swallowing sounds. We observed significantly better results in the maximum number of rhythmic swallows and the heart rate during the feeding session in late preterm infants. However, we found no differences between the groups in terms of time and milk intake.

Encouraging typical oral feeding is recommended as a key factor in determining when a patient can be discharged from the hospital and for supporting the developmental care in infants. It acts as an early indicator of neuromotor integrity and developmental progress. Supporting developmental care principles in the various interventions aimed at oral stimulation can assist infants in acquiring sucking abilities and improving oromotor coordination, ultimately facilitating earlier oral feeding and hastening the hospital discharge process. However, the feeding maturation process in preterm infants is complicated and involves multiple mechanisms, of which oral muscles and rhythmic swallows form only one. Oromotor skills can be improved through oromotor therapy, which specifically targets the strengthening of muscles engaged in oral activities like sucking and swallowing. By targeting these essential skills, oromotor therapy aims to improve the coordination and efficiency of feeding, promoting successful breastfeeding. Oromotor therapy has not been associated with any reported side effects or complications [3,26,27]. Ostadi et al. [28] enrolled 45 infants in a neonatal intensive care unit (NICU), with an average gestational age of 28.5 weeks and a birth weight of 1193 g, dividing them into three groups: one receiving non-nutritive sucking (NNS) exercises, another undergoing both NNS and swallowing exercises (SE), and a control group. Their study found that infants in the NNS or NNS + SE groups required less tube feeding upon discharge, and their feeding skill scores were higher than those of the control group. A meta-analysis suggests that NNS accelerates the achievement of full oral feeding, and sensorimotor interventions show promise in improving the sucking process [28]. The ESPGHAN Committee on Nutrition and Invited Experts recommends engaging in NNS prior to initiating oral feeding, as it is associated with reduced time to reach full oral feeding and shorter hospital stays [18]. Although some studies lack specific details about the stimulation program, oromotor therapy has shown positive effects on feeding skills in infants. The present study employed the KT method to stabilize and facilitate the feeding muscles, promoting the suck–swallow performance of the infants and resulting in developmental well-being in infancy.

In this study, we used the KT method to promote the sucking and swallowing performance of late preterm infants. There are numerous KT-related studies on children with torticollis, developmental delay, cerebral palsy, and swallowing difficulties. One case report mentioned that the KT application was used to support swallowing in a preterm infant at the 28th gestational week due to dysphagia at postmenstrual week 40, and successful results were obtained [13]. Lin et al. [13] proposed that KT adheres effectively to skeletal muscles, facilitating the activation of the orbicularis oris muscle for lip closure, the masseter muscle for jaw movement and chewing, and the mylohyoid muscle to elevate the hyoid bone. Moreover, KT may inhibit the activity of the sternohyoid muscle, enhancing sucking and swallowing functions. Jung et al. [19] proposed that taping might enhance oropharyngeal muscle thickness, presenting a potential therapeutic exercise for individuals with dysphagia following a stroke. Additionally, reports indicate that the application of KT causes a downward pull on the hyoid bone and larynx, thereby improving the activation of the suprahyoid muscle during swallowing in adults undergoing dysphagia rehabilitation [29]. A recent publication has also highlighted the effectiveness of the KT method in improving speech intelligibility and addressing drooling in children experiencing dysphagia [30]. We also found better results in the maximum number of rhythmic swallows of the infants and a lower heart rate, which shows more stability in swallowing during milk intake. Although the heart rate of the control group was higher, the heart rate of both groups was within the expected neonatal heart rate range. These results are the strength of our study and are very important in the feeding performance of preterm infants.

Several methods have been employed to assess oral feeding skills in infants, ranging from invasive measurements and non-invasive pressure assessments to the use of evaluation scales. Amaizu et al. [31] investigated the development of oral skills in 16 preterm infants by placing two catheters on the bottle nipple to assess sucking and using a small pressure drum to measure swallowing. Lagos et al. [32] utilized sonar Doppler to assess swallow sounds in both term and preterm newborns, finding it valuable for clinical evaluations. Geddes et al. [33] used ultrasound imaging in infants under 4 months old during breastfeeding, showing its effectiveness in detecting swallowing by visualizing the movement of the milk bolus through the pharyngeal area. Digital stethoscopes have also been utilized in the literature to record swallowing sounds. Anuk-Ince et al. [23] investigated swallowing sounds for assessing feeding maturation in preterm infants, basing feeding performance on data collected during audio recordings. Various parameters, such as the total number of swallows, maximum number of rhythmic swallows, resting intervals, and average times between intervals were generated for each assessment. They found that swallowing sounds could effectively assess feeding maturation in preterm infants during NICU follow-up. They also found that by using the swallow sound technique, they were able to observe that, as sucking and swallowing mature, the maximum number of rhythmic swallows increases.

Additionally, Ecevit et al. [34] found a positive correlation between feeding volume and total swallow count, swallow time, and maximum rhythmic swallow count, as recorded by a neonatal oral feeding monitor (NeoSAFE). More maximum rhythmic swallow patterns may indicate that infants with a developed swallowing function can handle larger feeding volumes. In our study, we similarly utilized swallowing sounds to measure the maximum number of rhythmic swallows during milk intake, and our result was higher in the KT group than in the control group.

Due to suck-and-swallow dysfunction, neonates may have difficulty breastfeeding or bottle feeding, which increases the risk of aspiration. In the literature, no study was found showing that KT application prevents aspiration in preterm infants. In our study, we also did not examine the aspiration of infants. Gülec et al. [35] investigated the effect of KT on stroke-related dysphagia and found effective results in aspiration scores. Further studies are needed to investigate the effect of KT on aspiration in preterm infants.

In our study, the feeding type of the infants was different between the groups. The number of infants feeding with breastmilk was greater in the control group, while the mixed feeding type was much more common in the study group. This difference may be due to underlying factors such as maternal choice, socio-economic status, healthcare advice, or study-related influences. However, this different feeding type may affect the results of the study. Therefore, further studies are needed using the same feeding types across the groups.

The number of studies using KT in the infant period is quite small when the literature is examined. Öhman [8] and Giray et al. [9] conducted studies on congenital muscular torticollis using KT and found positive effects. Recently, Nederifar et al. [36] conducted a study using the KT method in preterm infants; however, it is different from our research, as it was not a randomized trial and the outcome measure was POFRAS. They also found positive results for KT in preterm infants.

Our study has some limitations, including being a single-center study with a relatively small sample size. Further research with larger sample sizes and more diverse KT interventions is needed to gain a better understanding of improvements in oral feeding skills in preterm infants. Another limitation is that the Apgar scores of the infants in the control group were significantly lower than those in the study group. However, since all of the infants were clinically stable in terms of health status and nutrition, we do not think that the lower Apgar scores affected the results.

## 5. Conclusions

The application of the KT technique showed no adverse effects on preterm infants during the feeding skills intervention. The assessment using acoustic analysis demonstrated that the number of maximum rhythmic swallows was significantly higher and that the infants had a more mature swallowing function and better heart stabilization during feeding in the KT group. Further studies are warranted incorporating different application types and techniques with larger sample sizes, especially among preterm infants with an early gestational age in the NICU, to stabilize the suck and swallow muscles.

## Figures and Tables

**Table 1:** Clinical characteristics of the infants.

	KT Group X ± SD *n* = 37	Control Group X ± SD *n* = 37	*p* Value
Gestational age (weeks)	35.31 ± 0.82	35.29 ± 0.81	0.838
Birth weight (grams)	2330.56 ± 389.64	2426.21 ± 421.36	0.295
Gender			
Girls *n* (%)	25 (56.8)	17 (45.9)	0.329
Boys *n* (%)	19 (43.2)	20 (54.1)	
Apgar score			
1st min	8.18 ± 0.81	7.64 ± 0.91	0.002 *
5th min	9.34 ± 0.52	8.91 ± 0.64	0.003 *
Maternal age (years)	32.34 ± 4.19	32.75 ± 5.36	0.939
Twin pregnancy *n* (%)	24 (54.5)	26 (70.3)	0.707
Feeding type (%)			
Breastmilk	11 (25.0)	22 (59.5)	0.001 *
Formula	3 (6.8)	5 (13.5)	
Mixed	30 (68.2)	10 (27.0)	

* *p* < 0.05.

**Table 2:** Comparing oral feeding and swallowing parameters between the groups.

	KT Group X ± SD (*n* = 37)	Control X ± SD (*n* = 37)	*p* Value
Amount of milk intake (mL)			
*before KT*	16.81 ± 6.38	16.35 ± 7.0	0.837
*after KT*	17.71 ± 6.22	18.27 ± 7.58	0.606
*after 24 h of KT*	21.47 ±7.45	23.33 ± 10.64	0.381
Time for milk intake (s)			
*before KT*	121.70 ± 76.67	119.72 ± 1.64	0.045 *
*after KT*	112.38 ± 51.33	117.03 ± 16.52	0.114
*after 24 h of KT*	123.92 ± 36.77	120.00 ± 15.57	0.816
Maximum number of rhythmic swallows during milk intake (*n*)			
*before KT*	16.04 ± 10.32	14.80 ± 10.99	0.637
*after KT*	29.47 ± 20.16	14.53 ± 7.37	0.000 *
*after 24 h of KT*	37.14 ± 21.16	19.20 ± 8.87	0.000 *
Heart rate during milk intake (/min.)			
*before KT*	136.02 ± 10.32	141.37 ± 2.65	0.152
*after KT*	138.43 ± 8.54	141.67 ± 2.64	0.033 *
*after 24 h of KT*	135.85 ± 8.27	139.53 ± 3.21	0.021 *
Oxygen saturation during milk intake (%)			
*before KT*	96.84 ± 2.03	96.00 ± 1.22	0.015
*after KT*	96.40 ± 1.78	96.18 ± 1.22	0.677
*after 24 h of KT*	96.86 ± 2.10	96.43 ± 1.30	0.225
Number of days for transition to full oral feeding	2.64 ± 1.32	3.02 ± 2.36	0.924
Number of days in the hospital	5.50 ± 2.90	5.50 ± 4.60	0.373
Number of days taking oxygen	6.27 ± 17.01	2.14 ± 3.46	0.657

* *p* < 0.05, KT: Kinesio taping, mL: milliliter, s: second, *n*: number, min: minute.

## Data Availability

The data presented in this study are available in the article.

## Abbreviations

The following abbreviations are used in this manuscript:

DOAJ	Directory of open access journals
TLA	Three letter acronym
LD	Linear dichroism
